# Heart Failure Therapies following Acute Coronary Syndromes with Reduced Ejection Fraction: Data from the ACSIS Survey

**DOI:** 10.3390/jpm13061015

**Published:** 2023-06-19

**Authors:** Barak Zafrir, Tal Ovdat, Mahmood Abu Akel, Fadel Bahouth, Katia Orvin, Roy Beigel, Offer Amir, Gabby Elbaz-Greener

**Affiliations:** 1 Lady Davis Carmel Medical Center, Cardiology Department, Ruth and Bruce Rappaport Faculty of Medicine, Technion-Israel Institute of Technology, 7 Michal St., Haifa, Israel; dr.m7moud_akel@hotmail.com; 2 Leviev Heart Center, Sheba Medical Center, Tel Hashomer, Israel; tal.cohen@sheba.health.gov.il; 3 Cardiology Department, Bnai Zion Medical Center, Ruth and Bruce Rappaport Faculty of Medicine, Technion-Israel Institute of Technology, Haifa, Israel; 4 Rabin Medical Center, Cardiology Department, Sackler Faculty of Medicine, Tel Aviv University, Tel Aviv, Israel; katiaorvin@gmail.com; 5 Leviev Heart Center, Sheba Medical Center, Cardiovascular Division, Sackler Faculty of Medicine, Tel Aviv University, Tel Aviv, Israel; beigelr@yahoo.com; 6 Hadassah Medical Center, Faculty of Medicine, Heart Institute, Hebrew University of Jerusalem, Jerusalem, Israel; oamir@hadassah.org.il (O.A.); gabbyelbaz100@gmail.com (G.E.-G.)

**Keywords:** heart failure, myocardial infarction, left ventricular dysfunction, drug therapy

## Abstract

Background: Guideline-directed medical therapies for heart failure (HF) may benefit patients with reduced left ventricular ejection fraction (LVEF) following acute coronary syndromes (ACS). Few real-world data are available regarding the early implementation of HF therapies in patients with ACS and reduced LVEF. Methods: Data collected from the 2021 nationwide, prospective ACS Israeli Survey (ACSIS). Drug classes included: (a) angiotensin-converting enzyme inhibitors (ACEI), angiotensin receptor blockers (ARB) or angiotensin receptor-neprilysin inhibitors (ARNI); (b) beta-blockers; (c) mineralocorticoid receptor antagonist (MRA) and (d) sodium-glucose cotransporter-2 inhibitors (SGLT2I). The utilization of HF therapies at discharge or 90 days following ACS was analyzed in relation to LVEF [reduced ≤40% (*n* = 406) or mildly-reduced 41–49% (*n* = 255)] and short-term adverse outcomes. Results: History of HF, anterior wall myocardial infarction and Killip class II-IV (32% vs. 14% *p* < 0.001) were more prevalent in those with reduced compared to mildly-reduced LVEF. ACEI/ARB/ARNI and beta-blockers were used by the majority of patients in both LVEF groups, though ARNI was prescribed to only 3.9% (LVEF ≤ 40%). MRA was used by 42.9% and 12.2% of patients with LVEF ≤40% and 41–49%, respectively, and SGLT2I in about a quarter of both LVEF groups. Overall, ≥3 HF drug classes were documented in 44% of the patients. A trend towards higher rates of 90-day HF rehospitalizations, recurrent ACS or all-cause death was noted in those with reduced (7.6%) vs. mildly-reduced (3.7%) LVEF, *p* = 0.084. No association was observed between the number of HF drug classes or the use of ARNI and/or SGLT2I with adverse clinical outcomes. Conclusions: In current clinical practice, the majority of patients with reduced and mildly-reduced LVEF are treated by ACEI/ARB and beta-blockers early following ACS, whereas MRA is underutilized and the adoption of SGLT2I and ARNI is low. A greater number of therapeutic classes was not associated with reduced short-term rehospitalizations or mortality.

## 1. Introduction

The pharmacotherapy of heart failure (HF) with reduced left ventricular ejection fraction (LVEF) (HFrEF; ≤40%) is historically based on the modulation of the renin-angiotensin-aldosterone axis and the sympathetic nervous system [1]. Angiotensin-converting enzyme inhibitors (ACEI), angiotensin receptor blockers (ARB), beta-blockers and mineralocorticoid receptor antagonists (MRA), are the mainstay of treatment for HFrEF and were shown to improve patients’ symptoms, risk of HF hospitalizations and survival [2]. The drug management of HFrEF has progressed in recent years, with the introduction of novel evidence-based treatments with beneficial effects on cardiovascular outcomes, including angiotensin receptor-neprilysin inhibitor (ARNI) and sodium-glucose cotransporter-2 inhibitors (SGLT2I) [3,4,5]. Differing from HFrEF, the optimal treatment potential for patients with HF with mildly-reduced ejection fraction (HFmrEF; 41–49%) is less established, and based mainly on data from a subgroup analysis of prospective randomized controlled trials in HF patients with preserved ejection fraction (HFpEF), or studies that did not meet their primary endpoint [6]. Nevertheless, recent guidelines recommend initiating the four pillars of HF management in symptomatic patients with HFmrEF, with class IIb indication given to ACEI, ARB, ARNI, MRA and beta-blockers, whereas SGLT2I were given a class IIa indication as they were shown to reduce cardiovascular mortality and HF hospitalizations in HF patients across the spectrum of LVEF [7]. 

Patients with reduced LVEF following myocardial infarction (MI) are at an increased risk of adverse events such as death and recurrent hospitalizations due to exacerbation of HF [8]. The above evidence-based HF medications may also benefit patients early post-MI with evidence of systolic dysfunction by reducing left ventricular remodeling, even in the absence of acute HF [9]. Accordingly, evidence-based HF drug management is suggested in patients with Pre-HF (Stage B), such as those with recent MI and systolic LV dysfunction, in order to prevent symptomatic HF and reduce mortality [7]. However, although several drugs including beta-blockers, ACEI and MRA were proven to be effective at reducing cardiovascular risk in high-risk patients following MI, others such as ARNI did not show a conclusive positive effect in this setting, [10] whereas ongoing trials currently evaluate the potential role of SGLT2I in the post-MI population [11,12]. In this regard, it should be noted that initiation of evidence-based therapies before hospital discharge may be of importance because HF medications are less likely to be initiated ambulatory by primary care physicians, and the delay of disease-modifying therapies may potentially cause harm [13,14].

The aim of the present study was to investigate the early implementation of HF therapies in patients admitted with acute coronary syndrome (ACS) and evidence of reduced (≤40%) compared to mildly reduced (41–49%) LVEF. Furthermore, we wished to analyze the association between the utilization of evidence-based HF therapies as documented at discharge and 90-day post-hospitalization with short-term adverse cardiovascular outcomes and mortality.

## 2. Methods

### 2.1. ACSIS Survey

Data were retrieved from the Acute Coronary Syndrome Israeli Survey (ACSIS). The ACSIS is a national survey that is conducted over a 2-month period every 2–3 years. Data were collected prospectively from all consecutive patients hospitalized with a diagnosis of ACS in all 25 coronary care units and cardiology wards operating in Israel. Patient management was at the discretion of the treating physicians. Eligibility for the survey was validated before discharge. Discharge diagnoses were recorded as determined by the attending physicians based on clinical, electrocardiographic, echocardiographic, and biomarker criteria. Demographic, historical and clinical data, including medical management, were recorded on pre-specified e-forms by dedicated study personnel. The survey is governed and coordinated by the working group on acute cardiovascular care of the Israeli Heart Society in collaboration with the Israeli Center for Cardiovascular Research (ICCR). The data storage, maintenance, and processing are performed by the ICCR, which also reviews documents to ensure data validity and quality. Ninety-day clinical adverse outcomes and 1-year mortality were ascertained by hospital chart review, telephone contact, and use of the Israeli National Population Registry. The ACSIS was approved by the ethics committees of all participating centers and was conducted according to the principles expressed in the Declaration of Helsinki. All patients provided written informed consent for the collection of the data and subsequent analysis. Endpoints were prespecified by the ACSIS steering committee.

### 2.2. Study Population

**Figure 1:**
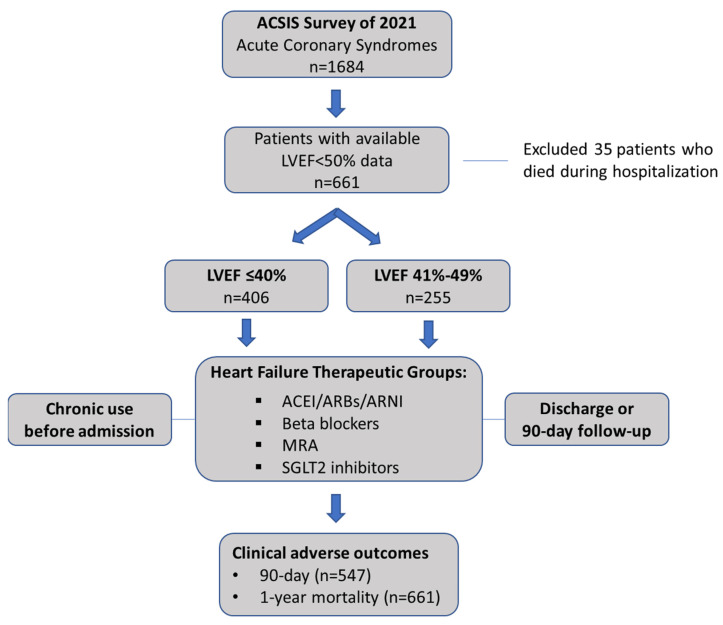
Study flowchart.

In the current study, the latest ACSIS survey performed during February–March 2021 was analyzed, in order to enable the assessment of novel evidence-based HF drug classes. The survey included 1684 consecutive patients with a discharge diagnosis of ACS, including unstable angina (UA), non-ST-segment elevation myocardial infarction (NSTEMI), and ST-segment elevation myocardial infarction (STEMI). We investigated 661 of the 1684 patients that had in-hospital echocardiographic evidence of reduced LVEF of less than 50% and survived the acute hospitalization. Participants were categorized as reduced LVEF ≤ 40% or mildly reduced LVEF 41–49%. The study flowchart is presented in Figure 1. 

Data on demographics, baseline characteristics, risk factors, medical history (prior coronary artery disease, history of HF, stroke, peripheral artery disease, and cancer), and medication history were prospectively collected. In addition, in-hospital information on ACS presentation, angiographic and revascularization data, use of cardiovascular procedures, in-hospital complications, laboratory values and pharmacologic management, were obtained until discharge. The drugs analyzed for the management of patients with reduced and mildly-reduced LVEF were categorized into four therapeutic groups: (a) beta-blockers, (b) ACEI, ARBs or ARNI, (c) MRA and (d) SGLT2I. In addition, the use of diuretics was documented. The utilization of medications was examined at several time points: (a) chronic use prior to the current admission, (b) medications initiated during the index hospitalization, and (c) at discharge or up to 90-day follow-up. 

Clinical adverse outcomes within 90 days post-hospitalization (available in 547 of the 661 patients) included recurrent ACS, stent thrombosis, urgent revascularization, stroke, HF hospitalization and aborted sudden cardiac death. In addition, cardiac and all-cause mortality of patients surviving hospitalization was documented up to 1-year from discharge in all study participants (*n* = 661). Referral to and participation in a cardiac rehabilitation program was also recorded. 

### 2.3. Data Analysis

Continuous variables are reported as means and standard deviation (±SD) or medians and interquartile range (IQR), as appropriate. Categorical variables are described as numbers and percentages. Study groups were compared using the chi-square test for categorical variables and Student’s *t*-test or Mann–Whitney Wilcoxon Test for continuous variables, according to the normality of distribution. HF therapeutic groups are presented according to time of documentation (pre-admission, in-hospital and discharge or 90-day follow-up) and LVEF groups (≤40% vs. 41–49%; i.e., reduced vs. mildly-reduced). A McNemar’s test was used to compare frequencies of HF therapeutic classes between the LVEF groups in two time points (pre-admission versus discharge or 90-day follow-up). Cardiovascular outcomes and mortality were analyzed in relation to LVEF groups, the number of therapeutic classes used and the use of ARNI and/or SGLT2Is. Pairwise comparisons between 0–1, 2 and 3–4 HF therapy groups were performed with Bonferroni’s correction for the 3 comparisons in each LVEF group. Overall, data had about 10% missing values and no imputation was performed; data on HF drug therapies were complete. All tests were conducted at a two-sided overall 5% significance level (α = 0.05). All statistical analyses were performed using R statistical software (R-studio, V.4.1.3; Vienna, Austria).

## 3. Results

### 3.1. Baseline and In-Hospital Characteristics

We analyzed 661 patients with ACS and an LVEF < 50% that were enrolled in the 2021 ACSIS survey and survived hospitalization. Of them, 406 (61.4%) had an LVEF ≤ 40% and 255 (38.6%) had an LVEF of 41–49%. The mean age of the study population was 64 ± 12 years and 82% were males. Patients’ baseline characteristics according to the LVEF group (≤40% vs. 41–49%) are presented in Table 1. Modifiable risk factors associated with atherosclerotic cardiovascular disease were comparable between both groups (hypertension 61%, dyslipidemia 66%, diabetes 43% and active smoking 47%). A history of HF was significantly more prevalent in patients with reduced versus mildly-reduced LVEF (15.3% vs. 7.1%, *p* = 0.002). Anterior location of MI on electrocardiogram was more typical in the reduced LVEF group, with the left anterior descending coronary artery the infarct-related artery in 75% of STEMI patients with an LVEF ≤ 40% compared to 45% in those with LVEF 41–49% (Supplemental Table S1). In-hospital complications are presented in Supplemental Table S2. ACS patients with reduced LVEF ≤ 40% had more symptomatic HF, with 32% defined as Killip class II-IV compared to 14% of those with LVEF 41–49%, *p* < 0.001. In addition, a trend towards higher rates of atrial fibrillation and acute renal failure was noted in the reduced LVEF group. 

### 3.2. Drug Therapy for Heart Failure

**Figure 2:**
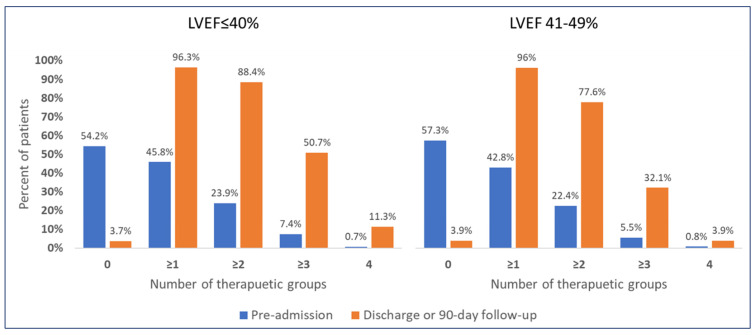
Number of heart failure therapeutic groups used before admission versus at discharge or 90-day follow-up, according to ejection fraction groups. *p* < 0.001 for comparison between LVEF ≤40% and 41–49%. Therapeutic groups include (a) ACEI or ARBs or ARNI, (b) beta-blockers, (c) MRA, (d) SGLT2I.

**Figure 3:**
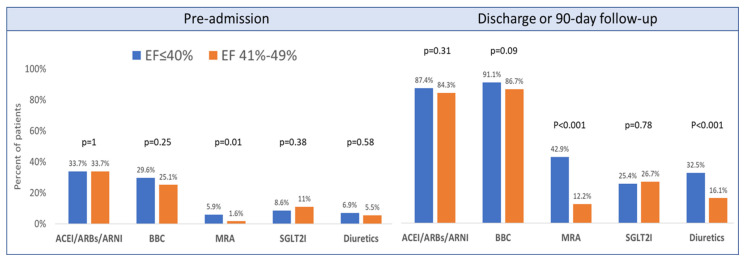
Drug therapies for heart failure, pre-admission and at discharge or 90-day follow-up, according to ejection fraction groups.

More than half of the patients presenting with ACS and reduced or mildly-reduced left ventricular systolic function were not treated by any of the HF drug classes before admission. At discharge or during 90-day follow-up, 84.3% of the patients were treated by two or more therapeutic classes, 43.6% by ≥3 drugs, and 8.5% by all four groups, with higher rates of utilization of 2–4 drug classes among patients with reduced compared to the mildly reduced LVEF group, *p* < 0.001 (Figure 2). A description of HF drug therapies used before admission, initiated during hospitalization, and prescribed at discharge or during 90-day follow-up, are presented in Table 2 and Figure 3. A significant increase in the utilization of all four HF therapeutic groups, as well as diuretics, was observed at discharge and short-term follow-up after hospitalization. ACEI/ARBs/ARNI and beta-blockers were used by the majority of patients in both LVEF groups. However, ARNI was given to only 3.9% of patients with LVEF ≤ 40%. MRA was documented in 42.9% of patients with LVEF ≤ 40% compared to 12.2% of those with LVEF 41–49%, *p* < 0.001. SGLT2I was similarly prescribed to about a quarter of patients with reduced and mildly-reduced LVEF at discharge or 90-day follow-up, mainly in patients with diabetes. Diuretics were utilized twice as much in those with reduced vs. mildly reduced LVEF (32.5% vs. 16.1%, *p* < 0.001).

ACEI, angiotensin-converting enzyme inhibitors; ARB, angiotensin receptor blockers; ARNI, angiotensin receptor neprilysin inhibitor; BBC, beta-blockers; EF, ejection fraction; MRA, mineralocorticoid receptor antagonist; SGLT2I, sodium-glucose cotransporter 2 inhibitors.

## 4. Clinical Outcomes following ACS

HF rehospitalizations during the 90-day follow-up occurred in 3.7% of patients with an LVEF ≤ 40% compared to 0.9% in those with an LVEF of 41–49%, *p* = 0.085 (Table 3). A trend towards a higher rate of a combined outcome (90-day HF rehospitalizations, recurrent ACS or all-cause death) was also noted (7.6% vs. 3.7%, *p* = 0.084). One-year mortality rates of patients surviving hospitalization were numerically higher in the reduced (4.2%) compared to the mildly-reduced (1.6%) LVEF group, but did not reach statistical significance, *p* = 0.101. No significant differences were observed in the rate of clinical adverse outcomes between 0–1, 2 and 3–4 HF therapeutic groups used at discharge or 90-day follow-up, in both LVEF groups (Supplemental Table S3). In addition, patients receiving ARNI and/or SGLT2I at discharge had similar short-term rehospitalization rates and mortality as compared with those without ARNI or SGLT2I (Supplemental Table S4).

Overall, 73% of the patients were referred to cardiac rehabilitation at discharge from hospitalization, and 34% of those with 90-day follow-up participated or were scheduled for an appointment in a rehabilitation center, with similar rates in those with reduced and mildly-reduced LVEF.

## 5. Discussion

In a contemporary nationwide survey of patients admitted with ACS and echocardiographic evidence of systolic left ventricular dysfunction, we have observed that the majority of patients with reduced or mildly-reduced LVEF surviving hospitalization received ACEI/ARBs and beta-blockers at discharge or during 90-days post hospitalization. Approximately one-third were treated by MRA and a quarter by SGLT2I, whereas the adoption of ARNI was particularly low (4% of those with LVEF ≤ 40%). Overall, the four pillars of HF management were documented early following ACS in 11.3% of patients with reduced and 3.9% of mildly-reduced LVEF. The utilization of MRA, ARNI, and diuretics was significantly lower in those with mildly-reduced LVEF. Although a trend for increased rehospitalizations and mortality was noted in patients with an LVEF ≤ 40% compared to 41–49%, no significant association was seen between the number of HF drug classes used and adverse clinical outcomes, in both LVEF groups, including in participants receiving ARNI and/or SGLT2I.

Among patients with acute MI, HF is a powerful predictor of death, and has important implications for medical treatment [15]. Post-MI HF is common and may develop in up to a third of older patients during the index MI event, with a frequency of 10% in HF hospitalizations within 1-year [16]. In the current study, a third of ACS patients with reduced LVEF that survived hospitalization were reported to present with Killip class II-IV, and therefore, were at a greater potential risk for developing chronic HF and mortality after discharge. In these patients, early initiation of disease-modifying HF therapies may be important in order to inhibit neurohormonal activation and prevent maladaptive myocardial remodeling that may precede chronic HF development [17].

Several post-MI trials of therapies with proven efficacy in patients with chronic HFrEF have demonstrated benefits also in the post-MI population with reduced LVEF. Among these therapies are beta-blockers, ACEI or ARBs and MRA [9]. However, these clinical trials often differed in their inclusion criteria regarding LVEF cutoff, the proximity to the acute MI event, and the requirement for HF signs and symptoms or increased levels of biomarkers [9]. Possible differences may, therefore, exist between chronic HF and early post-MI populations regarding drug management. Patients without current or prior symptoms or signs of HF but evidence of reduced left ventricular systolic function are classified as Pre-HF (stage B) [18]. These patients may be at increased risk of progression to symptomatic HF, and therefore, have an indication for specific treatments for asymptomatic left ventricular dysfunction in the acute phase, though whether they should be treated similarly to those with apparent HF (Stage C) is often debated and is still a subject to active research. ARNI, which has shown benefit in chronic HF patients with reduced LVEF, did not display similar benefit in the setting of acute MI in the recent PARADISE-MI trial [10]. In addition, SGLT2I that have demonstrated significant efficacy in improving outcomes of HF patients across all LVEF groups, have excluded patients with recent MI, and therefore, the potential efficacy and safety of SGLT2I early post-MI is yet to be proven in ongoing trials [11,12]. In this context, the recent EMMY trial findings are encouraging, demonstrating a greater reduction in NT-proBNP and improvement in functional and structural echocardiographic parameters in patients receiving empagliflozin early post-MI [19].

Recent ESC HF guidelines (2021) have changed the terminology of HF with mid-range LVEF to HF with mildly reduced LVEF (HFmrEF), as retrospective subgroup analysis of clinical trials and observational studies that included patients with LVEF 40–50% have shown some benefit from proven therapies for HFrEF [2]. In the era of early coronary revascularization, the number of patients with mildly reduced LVEF after acute MI is increasing, and ischemic heart disease is the leading etiology for HFmrEF, comparable to HFrEF [20]. In our cohort, similar rates of risk factors and comorbidities were seen in ACS patients with LVEF ≤40% and 41–49%, though as expected, patients with more reduced LVEF were reported to have higher rates of HF during hospitalization with a trend towards worse cardiovascular outcomes. Whether HFmrEF is a distinct entity or a transition phase between HFrEF and HFpEF, is controversial, as HFmrEF often has a complex pathophysiology and diverse clinical phenotypes [20]. The revised treatment recommendations for HFmrEF support providing drug therapy similar to HFrEF but with a weaker class of recommendation, except for SGLT2I and diuretics in congested patients [2,7]. However, the lack of outcome-proven therapies is reflected in the variability of HF medications prescribed to HFmrEF patients, who are often undertreated [21]. This was also evident in the current survey with the treatment of MRA that was ×3.5 times more prevalent in those with reduced compared to a mildly-reduced LVEF. In addition, reimbursement for HF medications often relies on LVEF qualification, and therefore, they are less attainable in patients with mildly reduced LVEF.

In the current survey, the implementation of ARNI early following ACS was low, reaching 4% in the LVEF ≤ 40% group. Contributing to the low utilization rate is the fact that ARNI is included in the national health basket in Israel only for chronic HF patients with LVEF ≤ 35% who are currently under treatment with ACEI/ARBs and beta-blockers. In addition, ARNI may not have been widely used as there is a lack of evidence on its beneficial effects early post-MI, [10] although exploratory analysis did show a reduction in total recurrent HF hospitalizations [22]. Of note, slow early adoption of ARNI was previously demonstrated in large cohorts of HF populations. In the United States Get with the Guidelines-Heart Failure (GWTG-HF) registry, only 2% of patients hospitalized for HFrEF were prescribed ARNI during the year following FDA approval [23]. Several years later, following label expansion for patients with HFmrEF and HFpEF, discharge prescriptions of ARNI among patients hospitalized for HF with LVEF 41–60% increased from 1.6% to 4.3% [24].

In the ACSIS survey, only a small minority of the ACS patients with systolic dysfunction have received all four HF medication classes at discharge or at 90 days following hospitalization, though a significantly higher percentage (44%) had documentation of ≥3 drug classes. This is in line with recent findings from a prospective, global study on hospitalized patients with acute HF, reporting the prescription of three classes of guideline-recommended drugs (ACEI/ARB/ARNI, beta-blockers and MRA), in only 37% of patients at discharge (41% in high-income countries) and 34% of survivors at 6 months follow-up [25]. Although differing in design and context of the study population, both studies highlight the underuse and suboptimal implementation of guideline-recommended effective medications. In addition, acute hospitalization may serve as a tool to increase downstream long-term use of evidence-based therapies, as discharge prescriptions for medications are strongly correlated with continued post-discharge adherence, whereas eligible patients without a discharge prescription are less likely to be initiated with drug therapy as outpatients [26]. Of note, recent studies in patients with acute HF demonstrated that in addition to a more intensive treatment strategy, a rapid follow-up after discharge from hospitalization with scheduled outpatient visits in a dedicated clinic led to a lower risk of cardiovascular events and rehospitalizations compared to usual care [27,28].

In the current analysis, no short-term survival benefit or reduced rehospitalization rates were observed with the increase in the number of HF drug classes used or the utilization of ARNI and/or SGLT2I at discharge or 90-day follow-up, in both LVEF groups. As not all patients presenting with ACS and reduced LVEF develop a clinical syndrome of HF complicating MI, it is possible that there is a less apparent benefit from specific HF therapies in the short-term post-hospitalization phase. Additional speculations for the lack of benefit of more intensive therapy in our study population include the small sample size and observational design, residual confounding factors, and the possibility of reverse causation, such as the prescription of a higher number of therapeutic classes to higher-risk patients.

## 6. Limitations

This study has several limitations. First, the number of patients participating in the survey and included in the current analysis was relatively low, as was the representation of women. Nevertheless, the survey is a prospective nationwide sample, evaluating consecutive patients and representing all Cardiology departments in the country. Second, LVEF was determined according to a single echocardiogram test performed during hospitalization, and no repeat assessment was available to examine whether systolic dysfunction following MI was partially reversible or transient. Third, we did not have information on electrolyte disturbances nor excluded patients with severe kidney dysfunction. Fourth, the ACSIS survey assessed HF drug treatment at several time points but did not include data on drug dosages, and therefore, we could not assess the titration of medications to optimal doses and physiological targets. Fifth, the collected data were recorded as determined by the attending physicians in each study site, and therefore, it may be subject to confounding factors. Finally, as this is a prospective, multicenter, observational survey, the association between treatment patterns and outcomes does not determine causality.

## 7. Conclusions

In a contemporary nationwide survey of patients admitted with an ACS, most patients with reduced or mildly-reduced LVEF were treated by ACEI/ARB and beta-blocker at discharge or 90-day post-hospitalization, though MRA was underutilized and the adoption of SGLT2I and ARNI was low. Only a minority of patients with systolic left ventricular dysfunction early following ACS were treated by all four pharmacological pillars of HF management. No association was noted between the number of HF therapeutic classes used or utilization of ARNI and/or SGLT2I with short-term rehospitalizations or mortality. The results emphasize the need for better optimization of drug therapy early following ACS in patients with reduced LVEF, and the requirement of further studies to affirm the benefit of combination HF therapies immediately post-MI in patients with systolic dysfunction that are at increased risk for progression to symptomatic HF.

## Figures and Tables

**Table 1:** Patients baseline characteristics.

Variable	Overall	LVEF ≤ 40%	LVEF 41–49%	*p*-Value
	*n* = 661	*n* = 406	*n* = 255	
Age, years (mean (SD))	63.97 (11.99)	64.50 (12.07)	63.12 (11.83)	0.150
Gender (male)	542 (82.0)	319 (78.6)	223 (87.5)	0.005
BMI (kg/m^2^) (median [IQR])	26.9 [24.5, 29.8]	26.8 [24.4, 29.7]	27.2 [24.5, 30.3]	0.260
Hypertension	403 (61.0)	252 (62.1)	151 (59.2)	0.516
Dyslipidemia	439 (66.5)	269 (66.3)	170 (66.9)	0.926
Diabetes mellitus	287 (43.4)	181 (44.6)	106 (41.6)	0.496
Current smokers	312 (47.2)	189 (46.6)	123 (48.2)	0.732
Past smokers	116 (17.5)	72 (17.7)	44 (17.3)	0.958
Chronic renal failure	64 (9.7)	44 (10.8)	20 (7.8)	0.258
Chronic Obstructive Pulmonary Disease	43 (6.5)	32 (7.9)	11 (4.3)	0.098
Any malignancy	36 (5.5)	21 (5.2)	15 (6.0)	0.800
Peripheral Vascular Disease	58 (8.8)	38 (9.4)	20 (7.9)	0.607
Prior Cerebrovascular Accident/Transient Ischemic Attack	60 (9.1)	43 (10.6)	17 (6.7)	0.116
History of Congestive Heart Failure	80 (12.1)	62 (15.3)	18 (7.1)	0.002
Prior Myocardial Infarction	236 (35.8)	154 (37.9)	82 (32.4)	0.176
Prior Coronary Artery Bypass Graft	43 (6.5)	27 (6.7)	16 (6.3)	0.977
Prior Percutaneous Coronary Intervention	230 (34.8)	149 (36.7)	81 (31.9)	0.239

**Table 2:** Drug therapy for heart failure.

	Chronic Use before Admission	Initiated during Hospitalization	Therapy at Discharge or at 90-Day Follow-Up
**EF ≤ 40% (*n* = 406)**			
ACEI (%)	82 (20.2%)	208 (51.2%)	260 (64%)
ARB (%)	53 (13.1%)	60 (14.8%)	103 (25.4%)
ARNI (%)	3 (0.7%)	4 (1%)	16 (3.9%)
MRA (%)	24 (5.9%)	143 (35.2%)	174 (42.9%)
Beta Blockers (%)	120 (29.6%)	270 (66.5%)	370 (91.1%)
SGLT2I (%)	35 (8.6%)	34 (8.4%)	103 (25.4%)
Diuretics (%)	28 (6.9%)	105 (25.9%)	132 (32.5%)
No. of HF therapeutic groups (median [IQR])	0 [0, 1]	2 [1, 3]	3 [2, 3]
**EF 41** **–49% (*n* = 255)**			
ACEI (%)	47 (18.4%)	134 (52.5%)	164 (64.3%)
ARB (%)	39 (15.3%)	26 (10.2%)	55 (21.6%)
ARNI (%)	0	0	1 (0.4%)
MRA (%)	4 (1.6%)	26 (10.2%)	31 (12.2%)
Beta Blockers (%)	64 (25.1%)	166 (65.1%)	221 (86.7%)
SGLT2I (%)	28 (11%)	26 (10.2%)	68 (26.7%)
Diuretics (%)	14 (5.5%)	26 (10.2%)	41 (16.1%)
No. of HF therapeutic groups (median [IQR])	0 [0, 1]	2 [1, 2]	2 [2, 3]

ACEI, angiotensin-converting enzyme inhibitors; ARB, angiotensin receptor blockers; ARNI, angiotensin receptor-neprilysin inhibitor; HF, heart failure; MRA, mineralocorticoid receptor antagonist; SGLT2I, sodium-glucose cotransporter-2 inhibitors.

**Table 3:** Adverse clinical outcomes, according to left ventricular ejection fraction group.

	Overall	LVEF ≤ 40%	LVEF 41–49%	*p*-Value
Patients with Available 90-Day Follow-Up				
*n*	547	328	219	
Rehospitalizations—ACS (cardiac, unscheduled) * (%)	12 (2.2)	9 (2.7)	3 (1.4)	0.437
Rehospitalizations—CHF (%)	14 (2.6)	12 (3.7)	2 (0.9)	0.085
ACS (UA/NSTEMI/STEMI/ Stent thrombosis) (%)	19 (3.5)	14 (4.3)	5 (2.3)	0.315
Aborted SCD (%)	1 (0.2)	1 (0.3)	0 (0.0)	1.000
90-day any mortality (%)	2 (0.4)	1 (0.3)	1 (0.5)	1.000
Combined outcome 90-days (CHF, ACS, any death) (%)	33 (6.0)	25 (7.6)	8 (3.7)	0.084
Referral to rehabilitation (%)	388 (72.8)	234 (73.6)	154 (71.6)	0.690
Participation in rehabilitation (or scheduled) (%)	175 (34.1)	106 (34.2)	69 (34.0)	1.000
**All patients**				
***n***	**661**	**406**	**255**	
30-day MACE ** (%)	52 (7.9)	34 (8.4)	18 (7.1)	0.643
1-year mortality *** (%)	21 (3.2)	17 (4.2)	4 (1.6)	0.101

* Refers to the first re-hospitalization only. ** MACE (30 days: death/UA/MI/CVA/stent thrombosis/urgent revascularization). *** Excluding in-hospital death. ACS, acute coronary syndrome; CHF, congestive heart failure; CVA, cerebrovascular accident; NSTEMI, non-ST segment myocardial infarction; SCD, sudden cardiac death; STEMI, ST-segment myocardial infarction; UA, unstable angina.

## Data Availability

Not applicable.

## Supplementary Materials

The following supporting information can be downloaded at: https://www.mdpi.com/article/10.3390/jpm13061015/s1, Table S1: In-hospital characteristics of study population, Table S2: In-hospital complications of study population, Table S3. Clinical adverse outcomes post-discharge, according to the number of heart failure therapeutic groups, Table S4: Clinical outcomes according to the use of ARNI or SGLT2I at discharge or 90-Days follow-up.
