# Supplementary material for: Heart Failure Therapies following Acute Coronary Syndromes with Reduced Ejection Fraction: Data from the ACSIS Survey
Source: J Pers Med. 2023 Jun 19;13(6):1015. doi: 10.3390/jpm13061015 (PMC10304454; doi:10.3390/jpm13061015)
Supplement: Supplementary file 1 [file jpm-13-01015-s001.zip › jpm-2416373-supplementary.pdf]

Supplemental Data

Heart failure therapies following acute coronary syndromes with reduced ejection fraction: Data from the ACSIS survey

Supplemental Table S1: In-hospital characteristics of study population

Variable	Overall n=661	LVEF≤40% n=406	LVEF 41-49% n=255	P value	Missing data (%)
In-hospital LVEF (median [IQR])	40 [35, 45]	35 [33, 40]	45 [45, 45]	<0.001	0
Discharge diagnosis:				0.546	0
NSTEMI	286 (43.3)	175 (43.1)	111 (43.5)		
STEMI	355 (53.7)	221 (54.4)	134 (52.5)		
UA	20 (3.0)	10 (2.5)	10 (3.9)		
Anterior STEMI location (ECG)	223 (62.8)	166 (75.1)	57 (42.5)	<0.001	
CABG (till hospital discharge)	44 (6.7)	27 (6.7)	17 (6.7)	1.000	0
AICD/CRT	10 (1.5)	9 (2.2)	1 (0.4)	0.123	0
Intra-aortic Balloon (IABP)	15 (2.3)	11 (2.7)	4 (1.6)	0.487	
**STEMI patients (n)**	**355**	**221**	**134**		
Angiography followed by:				0.526	8.2
Primary PCI	321 (98.5)	197 (98.0)	124 (99.2)		
Urgent CABG	3 (0.9)	2 (1.0)	1 (0.8)		
No intervention	2 (0.6)	2 (1.0)	0 (0.0)		
Infarcted related artery:				<0.001	9.3
LMCA	2 (0.6)	2 (1.0)	0 (0.0)		
LAD	206 (64.0)	151 (75.5)	55 (45.1)		
LCX	48 (14.9)	20 (10.0)	28 (23.0)		
RCA	63 (19.6)	26 (13.0)	37 (30.3)		
Surgical graft	3 (0.9)	1 (0.5)	2 (1.6)		
Number of diseased vessels:				0.292	8.5
No vessels	3 (0.9)	2 (1.0)	1 (0.8)		
1 vessel	131 (40.3)	80 (40.0)	51 (40.8)		
2 vessels	117 (36.0)	66 (33.0)	51 (40.8)		
3 vessels	74 (22.8)	52 (26.0)	22 (17.6)		
TIMI grade flow before revascularization (median [IQR])	0 [0, 2]	0 [0, 1]	0 [0, 2]	0.372	11.3
PCI for non-infarct artery	37 (11.4)	26 (12.9)	11 (8.9)	0.359	8.7

AICD, Automatic implantable cardioverter defibrillator; CABG, coronary artery bypass graft; CRT, cardiac resynchronization therapy; ECG, electrocardiography; IQR, interquartile range; LAD, left anterior descending coronary artery; LCX, left circumflex coronary artery; LMCA, left main coronary artery; LVEF, left ventricle ejection fraction; NSTEMI, non-ST segment elevation myocardial infarction; PCI, percutaneous coronary intervention; STEMI, ST-segment elevation myocardial infarction; SVG, saphenous vein graft; UA, unstable angina; RCA, right coronary artery

Supplemental Table S2: In-hospital complications of study population

Variable	Overall<br>661	LVEF≤40%<br>406	LVEF 41%-49%<br>255	P value
CHF mild-moderate (Killip II)	109 (16.5)	81 (20.0)	28 (11.0)	0.004
Pulmonary edema (Killip III)	40 (6.1)	36 (8.9)	4 (1.6)	<0.001
Cardiogenic shock (Killip IV)	20 (3.0)	16 (4.0)	4 (1.6)	0.134
Hemodynamically significant right Ventricular infarction	8 (1.2)	7 (1.7)	1 (0.4)	0.248
Post MI angina/re-ischemia	6 (0.9)	3 (0.7)	3 (1.2)	0.872
Stent thrombosis (definite/probable/possible)	5 (0.8)	4 (1.0)	1 (0.4)	0.695
MR moderate - severe	17 (2.6)	13 (3.2)	4 (1.6)	0.299
Pericarditis	7 (1.1)	6 (1.5)	1 (0.4)	0.349
Sustained VT (>125 bpm)	8 (1.2)	7 (1.7)	1 (0.4)	0.246
Primary VF	12 (1.8)	6 (1.5)	6 (2.4)	0.602
Secondary VF	3 (0.5)	2 (0.5)	1 (0.4)	1.000
New Atrial Fibrillation.	33 (5.0)	26 (6.4)	7 (2.7)	0.055
High degree (2nd or 3rd) AVB	4 (0.6)	2 (0.5)	2 (0.8)	1.000
Asystole	5 (0.8)	3 (0.7)	2 (0.8)	1.000
Stroke	4 (0.6)	3 (0.7)	1 (0.4)	0.965
Acute renal failure	55 (8.4)	41 (10.1)	14 (5.5)	0.051
Bleeding	18 (2.7)	15 (3.7)	3 (1.2)	0.090
Free wall rupture	1 (0.2)	1 (0.2)	0 (0.0)	1.000

AVB, atrioventricular block; CHF, congestive heart failure; MI, myocardial infarction; MR, mitral regurgitation, VF, ventricular fibrillation; VT, ventricular tachycardia

Supplemental Table S3: Clinical adverse outcomes post-discharge, according to the number of heart failure therapeutic groups

	EF≤40%			EF 41%-49%		
**No. of therapeutic groups:**	**0-1**	**2**	**3-4**	**0-1**	**2**	**3-4**
**Patients with 90-day follow-up (n=547)**	31	130	167	51	93	75
Rehospitalizations - ACS (cardiac, unscheduled) * (%)	0	6 (4.6%)	3 (1.8%)	0	0	3 (4%)
Rehospitalizations - HF (%)	2 (6.9%)	2 (1.6%)	8 (4.8%)	0	1 (1.1%)	1 (1.3%)
ACS (UA/NSTEMI/STEMI/ Stent thrombosis) (%)	0	8 (6.2%)	6 (3.6%)	0	2 (2.2%)	3 (4%)
Aborted SCD (%)	0	1 (0.8%)	0	0	0	0
90-day all-causes mortality (%)	0	1 (0.8%)	0	1 (2%)	0	0
Combined outcome 90-days (HF, ACS, any death) (%)	2 (6.5%)	9 (6.9%)	14 (8.4%)	1 (2%)	3 (3.2%)	4 (5.3%)
Referral to rehabilitation (%)	19 (67.9%)	94 (75.2%)	121 (73.3%)	33 (67.3%)	73 (78.5%)	48 (65.8%)
Participation in rehabilitation (or scheduled) (%)	9 (33.3%)	44 (36.4%)	53 (32.7%)	11 (24.4%)	37 (42.5%)	21 (29.6%)
**All patients (n=661)**	47	153	206	57	116	82
30-day MACE**(%)	2 (4.3%)	14 (9.2%)	18 (8.7%)	4 (7%)	8 (6.9%)	6 (7.3%)
1-year mortality*** (%)	3 (6.4%)	7 (4.6%)	7 (3.4%)	1 (1.8%)	2 (1.7%)	1 (1.2%)

Stratified by number of HF therapeutic groups [(a) any ACEI or ARB or ARNI, (b) MRA, (c) SGLT2I, (d) Beta blockers], prescribed at discharge or 90-day follow-up. All pairwise comparisons were non-significant

* Refers to the first re-hospitalization only
** MACE (30 days: death/UA/MI/CVA/stent thrombosis/urgent revascularization)
*** Excluding in-hospital death

ACS, acute coronary syndrome; CVA, cerebrovascular accident; HF, heart failure; MACE, major adverse cardiovascular events; NSTEMI, non-ST segment elevation myocardial infarction; SCD, sudden cardiac death; STEMI, ST-segment elevation myocardial infarction; UA, unstable angina

Supplemental Table S4: Clinical outcomes according to the use of ARNI or SGLT2I at discharge or 90-Days follow-up

	Overall	ARNI/SGLT2I No	ARNI/SGLT2I Yes	P value
**<u>Patients with available 90-day follow-up*</u>**				
**n**	547	387	160	
Rehospitalizations - ACS (cardiac, unscheduled) * (%)	12 (2.2%)	9 (2.3%)	3 (1.9%)	0.995
Rehospitalizations - HF (%)	14 (2.6%)	10 (2.6%)	4 (2.5%)	1
ACS (UA/NSTEMI/STEMI/ Stent thrombosis) (%)	19 (3.5%)	13 (3.4%)	6 (3.8%)	1
Aborted SCD (%)	1 (0.2%)	1 (0.3%)	0	1
90-day any mortality (%)	2 (0.4%)	2 (0.5%)	0	0.895
Combined outcome 90-days (HF, ACS, any death) (%)	33 (6%)	23 (5.9%)	10 (6.2%)	1
**<u>All patients</u>**				
	661	478	163	
30-day MACE** (%)	52 (7.9%)	37 (7.7%)	15 (8.2%)	0.973
1-year mortality*** (%)	21 (3.2%)	15 (3.1%)	6 (3.3%)	1

* Refers to the first re-hospitalization only
** MACE (30 days: death/UA/MI/CVA/stent thrombosis/urgent revascularization)
*** Excluding in-hospital death

ACS, acute coronary syndrome; CVA, cerebrovascular accident; HF, heart failure; MACE, major adverse cardiovascular events; NSTEMI, non-ST segment elevation myocardial infarction; SCD, sudden cardiac death; STEMI, ST-segment elevation myocardial infarction; UA, unstable angina
